# Histological characterization of the lateral root primordium development in rice

**DOI:** 10.1186/s40529-014-0042-x

**Published:** 2014-05-10

**Authors:** Jun Ni, Yan-Xia Shen, Yan-Yan Zhang, Yu Liu

**Affiliations:** 1 College of Life and Environmental Sciences, Hangzhou Normal University, Hangzhou, China; 2 State Key Laboratory of Plant Physiology and Biochemistry, College of Life Science, Zhejiang University, Hangzhou, China

**Keywords:** Rice, Lateral root primordium, Auxin

## Abstract

**Background:**

Lateral root constitutes an important part of root system either in tap root plants or fibrous root plants. The development of lateral root primordium (LRP) in *Arabidopsis*, which has a tap root system with simple radial structure of primary root, has been well characterized. However, limited knowledge has been acquired on the plants with fibrous root system, such as rice. This is mainly due to their multiple cell layers coated on root, which disturb the observation of LRP.

**Results:**

We used an easy and quick method to strip the epidermal and cortex tissues of primary root so that the LRP can be easily observed under microscope. In this way, we observed the developmental processes of LRP in rice. In addition, we described the expression dynamics of several root development related genes, especially *OsPINs* (*PIN-FORWMED*)*,* during the process of LRP development.

**Conclusions:**

We reported an easy and quick method for LRP observation in rice and suggested a “fountain” model of auxin transport in LRP of rice, which is similar with that in *Arabidopsis*.

**Electronic supplementary material:**

The online version of this article (doi:10.1186/s40529-014-0042-x) contains supplementary material, which is available to authorized users.

## Background

Plant root is an important organ for nutrient and water uptake and provides physical support for plant growth. Lateral roots sprout horizontally from the primary root and largely increase surface area of root system to contact with soil. They constitute the major functional part of the root system.

The development of LRP in *Arabidopsis* has been well documented. The process can be divided into eight stages according to specific anatomical characteristics (Malamy and Benfey [1997]). Briefly, the LRP is initiated from the anticlinal divisions of pericycle founder cells (stage I). Then the central short daughter cells divide periclinally to form an inner layer and outer layer (stage II). After series of subsequent divisions (stages III to VII), the LRP finally emerges from the parent root (Casimiro et al. [2003]). In the case of cereal plant, such as rice, the situation seems quite different. In rice, the LRP can be initiated from both pericycle and endodermis (Kawata and Shibayama [1965]), while in *Arabidopsis*, only pericycle cells contribute to the initiation of LRP (Dubrovsky et al. [2001]). In rice, there are several layers of epidermis and ground tissue structure covering the pericycle. They are composed of one layer each of epidermis, exodermis, sclerenchyma and five layers of cortex (Rebouillat et al. [2009]). This complex radial structure of primary roots makes it difficult to clearly observe the LRP through microscope.

In this study, we established an easy and quick method to observe the LRP development in rice. For the first time, we described the expression patterns of several developmentally related genes, especially *OsPINs*, during the process of LRP development. We proposed a “fountain” model of auxin transportation in LRP of rice. In this model, auxin is firstly redirected from parent primary root to the LRP via *OsPIN1c*, and then transported to the root tip via *OsPIN1b*, where the auxin is accumulated. This process is proved by the GUS (β-glucuronidase) activity of *DR5::GUS*. In the root tip, part of auxin is retrieved by *OsPIN2* mediated auxin transport.

## Results and discussion

**Figure 1:**
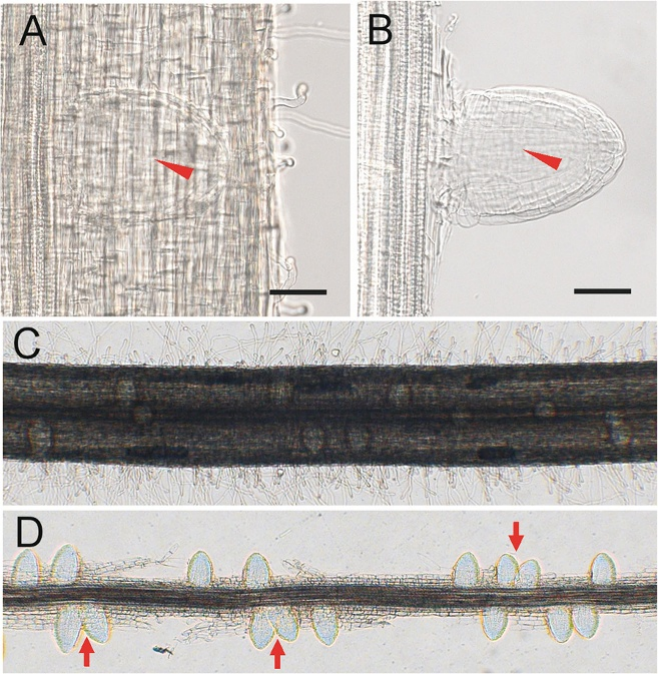
**Dissection of the epidermal and ground tissues to uncover the LRP in rice. (A)** LRP was covered by epidermal and ground tissues which disturb the observation greatly. Arrow head indicates the LRP, bar = 50 μm. **(B)** After dissection of covered layers, the LRP can be clearly observed under the microscope. Arrow head indicates the LRP, bar = 50 μm. **(C)** The arrangement of LRPs along the central cylinder cannot clearly seen in normal conditions. **(D)** After the removal of covered layers, the arrangement pattern and the number of LRPs can be seen clearly. Arrows indicate very closed LRP.

Because of the epidermal and the ground tissues, which are composed of exodermis, sclerenchyma, cortex and endodermis, heavily cover the LRP on root of rice (Rebouillat et al. [2009]), it is difficult to clearly observe the LRP through microscope (Figure 1A). To cope with this problem, we dissected the epidermal and ground tissues under the stereomicroscope to expose the LRP. In this way, the structure of LRP can be seen clearly under the microscope (Figure 1B). It was relatively hard to exactly count the number of LRPs along a segment of root (Figure 1C). In our experiment, after dissecting the epidermal and ground tissues, we can easily count the number of LRPs. Besides, the distribution pattern of LRPs along the central cylinder can also be observed. In *Arabidopsis*, the LRPs are allocated along the parent root in a regular left-right alternating pattern (De Smet et al. [2007]; Moreno-Risueno et al. [2010]). However, there was no obvious left-right alternating pattern observed in rice (Figure 1D). This implies that different regulation systems for LRP initiation exist between rice and Arabidopsis.

### The developmental characterization of LRP in rice

**Figure 2:**
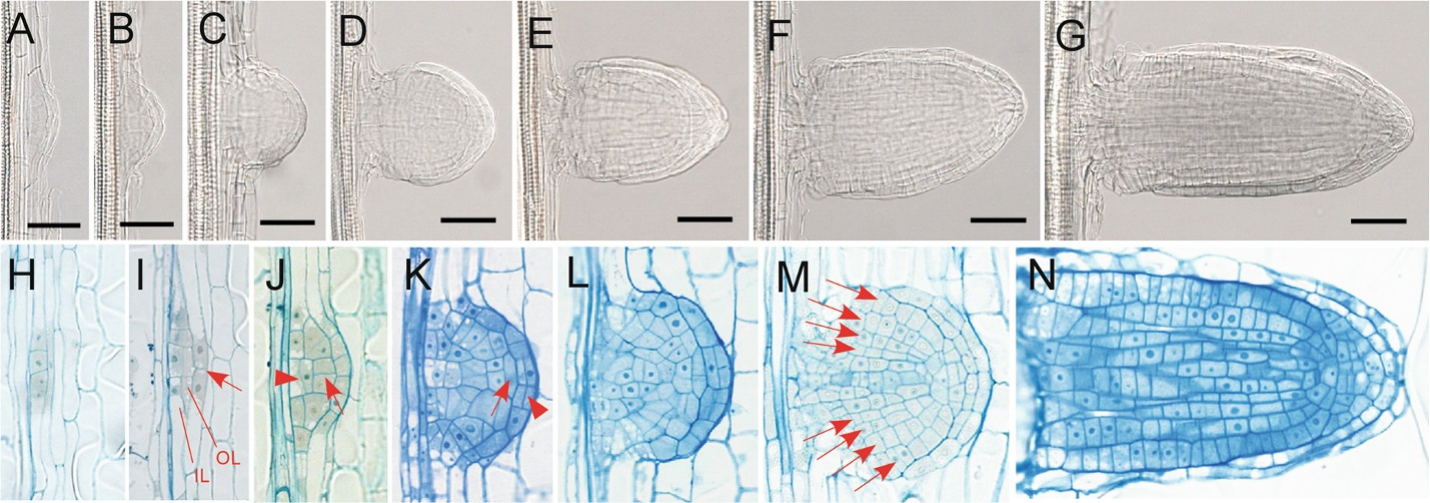
**The process of LRP development in rice. (A)-(G)** The observation of LRP development in rice using our easy quick method. **(G)** The emerged lateral root. Bars = 50 μm. **(H)-(M)** Sections of different stages of LRP. **(H)** Anticlinal cell divisions in pericycle. **(I)** Periclinal cell divisions in the center of LRP, resulting in two cell layers, inner layer (IL) and outer layer (OL). Arrow indicates anticlinal cell division in the endodermis. **(J)** Further development of LRP. Arrow head indicates periclinal cell division in the IL and arrow indicates anticlinal cell division in the OL. **(K)** The radial pattern begins to appear in the LRP. Arrow indicates the lens shaped cell which would finally form the root cap of lateral root. Arrow head indicates periclinal cell division in the central shelter. **(L)** and **(M)** LRP begins to resemble the mature lateral root tip. Arrows in **(M)** indicate 4 cell layers surrounding the stellar tissue. **(N)** Longitudinal section of emerged mature lateral root. The outmost shelter still exists around the root.

By this method above, the process of LRP development in rice can be clearly observed and characterized. The first scene of LRP initiation observed by this method is the transverse expansion of a specific root tissue, which then formed a dome-shaped LRP (Figure 2A). The dome-shaped LRP continues to grow and forms a hemispherical LRP (Figure 2B,C). After that, the LRP begins to expand transversely to form a shape of mushroom (Figure 2D,E). Then, it grows longitudinally to form a mature lateral root (Figure 2F,G).

To link the process of morphological changes described above to detailed cellular behavior during LRP development, longitudinal sections of LRPs were investigated. At the beginning of LRP development, increased anticlinal divisions are clearly seen in pericycle, resulting in short pericycle cells (Figure 2H). After several rounds of anticlinal divisions in pericycle, periclinal divisions occur in the center of LRP, resulting in two layers, outer layer (OL) and inner layer (IL) as described in Malamy and Benfey ([1997]). At the same time, the endodermis begins to divide anticlinally which will form a shelter cover the LRP (Figure 2I). The central IL cell divides periclinally, while the OL cell divides anticlinally to form small cuboidal cells. At the same time, the endodermis cells continue to divide anticlinally and the dome shaped LRP begins to appear (Figure 2J). At a certain stage of LRP development, several events appear to occur at approximately the same time. (1) The radial pattern begins to appear and the IL cells develop to form the stele, while the OL form the rest of the tissues. (2) A lens shaped cell appears in the tip of the LRP, which would finally form the root cap of lateral root. (3) The central shelter cells divide periclinally to form two layers of shelter. This shelter will cover the LRP and stop further dividing (Figure 2K). As the LRP continues to develop, LRP begins to resemble the mature lateral root tip. A core of presumptive stellar tissue is surrounded by 4 cell layers corresponding to epidermis, exodermis, sclerenchyma and endodermis as described by Rebouillat et al. ([2009]). The lens shaped cell also divides to form a potential root cap at the tip of LRP (Figure 2L,M). At the later stages of LRP development, the mushroom shaped primordium can be observed by the methylene blue staining (Figure 2M). At those stages, the cell arrangement of the LRP was very similar to that of the mature lateral root (Figure 2N). These results above showed that, first, the basic process of LRP development in rice is similar to that of *Arabidopsis* in the cellular level, and more importantly, we connect our morphological descriptions to the cellular organization of LRP which has been well characterized.

### Auxin distribution and gene expression patterns in the process of LRP development in rice

**Figure 3:**
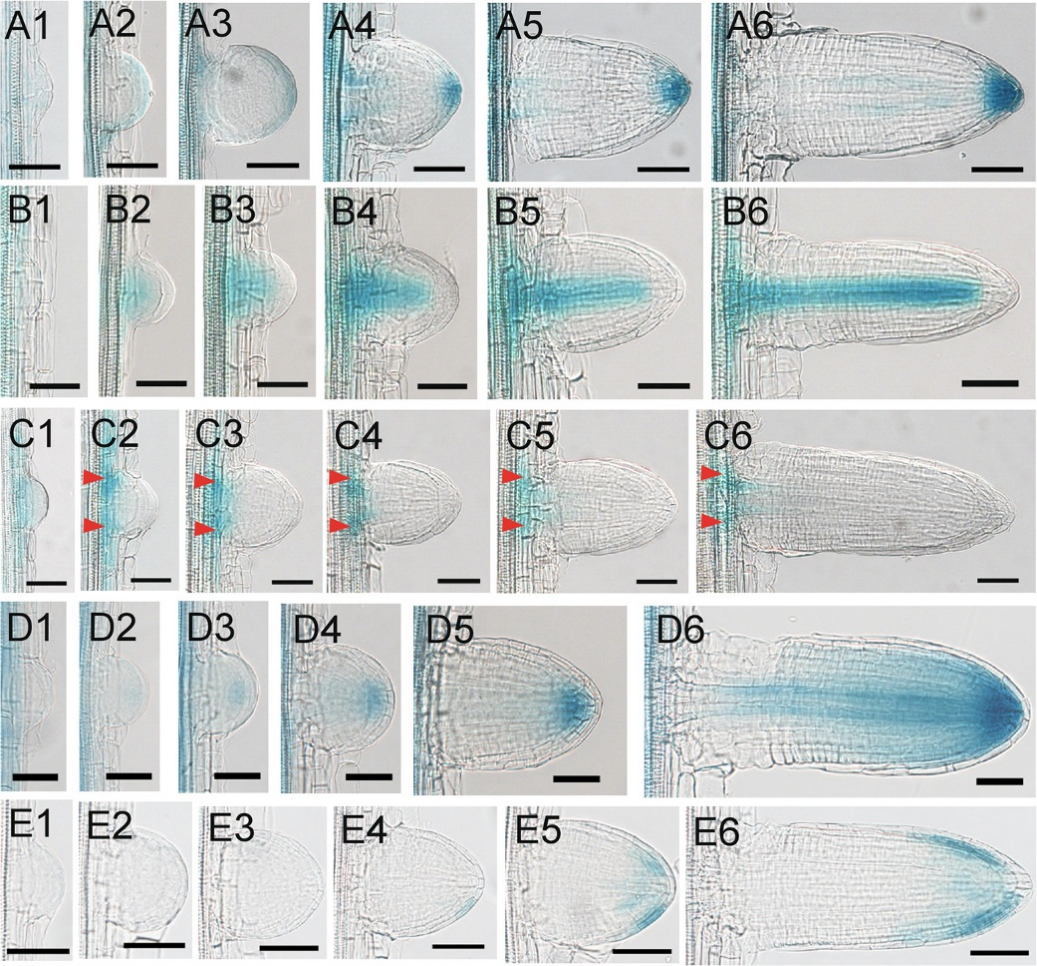
**Gene expressions in the process of LRP development in rice. (A1)-(A6)** The expressions of *DR5::GUS* during the development of LRP in rice. **(B1)-(B6)** The expressions of *OsSHR1::GUS* during the development of LRP in rice. **(C1)-(C6)** The expressions of *OsPIN1c::GUS* during the development of LRP in rice. Arrow heads indicate the expression peaks at the base of the primordia. **(D1)-(D6)** The expressions of *OsPIN1b::GUS* during the development of LRP in rice. **(E1)-(E6)** the expressions of *OsPIN2::GUS* during the development of LRP in rice. **(A6), (B6), (C6), (D6)** and **(E6)** are the emerged lateral roots. Bars = 50 μm.

The synthetic auxin responsive promoter *DR5* has been used as a tool for monitoring auxin response *in planta* (Ulmasov et al. [1997]). It has been shown that the activity of the reporter correlates to auxin content in roots (Benkova et al. [2003]; Casimiro et al. [2001]). We examined auxin response during the development of LRP in rice using *DR5::GUS* transgenic lines. GUS activity was detected at very early stages of development (Figure 3A1). When the LRP developed to a hemispherical shape, the GUS staining was observed uniformly in the peripheral of the LRP (Figure 3A2). After that, the GUS staining converged gradually to the central cells in the tip (Figure 3A3). During the progress to later stages, GUS activity was gradually enhanced on the tip of LRP (Figure 3A4-A6). The *DR5* activity during LRP development was similar to that in *Arabidopsis* at least in later stages (Benkova et al. [2003]). This indicates that auxin plays similar roles in the development of LRP in rice and *Arabidopsis*.

*OsSHR1 (SHORT-ROOT 1)* was reported to express in the stele of primary roots (Cui et al. [2007]). We examined its expression in the developing LRPs. *OsSHR1* was initially observed to express in the center of hemispherical shaped primordium (Figure 3B2, B3). As long as the LRP growing, *OsSHR1* was expressed in the central stele of the primordiun, which was very similar to its expression in the primary root (Figure 3B4-B6). These results also indicated that the identity of stele was existed at the very early stage of LRP development.

*OsPIN1c* (formerly named *OsPIN4*) was reported to express in the developing LRP (Wang et al. [2009]). We examined its expression in more detail during LRP development based on the method we developed. *OsPIN1c* was expressed uniformly in the early stage of LRP (Figure 3C1). Gradually, two peaks appeared in the base of LRP and its expression gradually converged to the two peaks (Figure 3C2-C6). From its expression pattern, we proposed that OsPIN1c may function in the junction of LRP and its parent stele to transport auxin between these two parts. We noticed that the two expression peaks were initially found in the boundary of LRP, and later in the central of LRP base, which indicates a transverse expansion in the LRP development.

*OsPIN1b* was reported to express in the root cap and the stele in the primary root (Wang et al. [2009]). We examined its expression in the developing LRP. However, we did not detect the expression of *OsPIN1b* at the very early stage of LRP development (Figure 3D1). When the LRP developed to a hemispherical shape, a weak GUS activity was detected in the center of LRP, indicating a weak expression of *OsPIN1b* at this stage (Figure 3D2). As the LRP developed, the GUS staining was clearly detected in the root cap of LRP (Figure 3D3-D5). Interestingly, we did not detect the GUS staining in the stele even though the stele identity was already existed based on the expression patterns of *OsSHR1*. The expression of *OsPIN1b* in the stele was not detected until the emergence of LRP (Figure 3D6). At this stage, the expression pattern of *OsPIN1b* was very similar with that in the primary root (Wang et al. [2009]).

*OsPIN2* was reported to express in the lateral root cap of primary root (Wang et al. [2009]). We examined its expression in the developing LRP. *OsPIN2* was not expressed at the early stages of LRP development (Figure 3E1-E3), which was similar to its ortholog *PIN2* in *Arabidopsis* (Benkova et al. [2003]). We did not observe the GUS staining until very late stage of LRP development (Figure 3E4, E5). After emergence, *OsPIN2* was expressed in margin of the shelter and the root cap in the lateral root, but not in the center of shelter or root cap (Figure 3E6). Its expression pattern in the lateral root was also very similar with that in the primary root (Wang et al. [2009]).

In this paper, we reported an easy and quick method for LRP observation in rice. A model was proposed in rice for LRP development. Initially, the LRP expands radically to form a dome shaped LRP. Then the dome shaped LRP further grows to a hemispherical shape. Later the LRP begins to expend transversely to form a shape of mushroom and after that, the LRP grows longitudinally to form a mature lateral root. In addition we also investigated the cellular bases of the morphological changes of LRP. Importantly, using this method, we described the expression dynamic patterns of several genes, especially *OsPINs,* during the rice LRP development for the first time. The PIN proteins are transporters acting in the efflux of auxin from cells. They have specific developmental roles that largely determined by their highly specific tissue expression (Krecek et al. [2009]). AtPIN1 mainly residues at the basal end of the vascular cells in root (Blilou et al. [2005]). AtPIN2 localizes apically in epidermal and lateral root cap cells and predominantly basally in cortical cells in the root tip, mediating the auxin maximum and auxin redistribution for root gravitropism (Muller et al. [1998]; Blilou et al. [2005]). In *Arabidopsis*, the LRP development was associated with an auxin gradient with a peak at the primordum tip. This gradient depends on differentially expressed PINs (Benkova et al. [2003]). In rice, OsPIN1b has been reported to play an important role in crown root emergence and tiller development (Xu et al. [2005]). Over-expression of *OsPIN2* led to a shorter plant hight, more tillers and a larger angle compared with wild type (Chen et al. [2012]). However, the roles of OsPINs in the development of LRP in rice have not been investigated. From the expression patterns of *DR5* and *OsPINs*, we suggested a “fountain” model for auxin transport in the LRP of rice, which was similar to *Arabidopsis* (Benkova et al. [2003]). Auxin is firstly redirected from parent primary root to the LRP via OsPIN1c. Then, auxin transport is mediated by the OsPIN1b to the root tip, where the auxin is accumulated. It can be proved by the staining of *DR5::GUS*. From here, part of the auxin is retrieved by OsPIN2 mediated auxin transport.

## Conclusions

We reported an easy and quick method for LRP observation in rice and a model was proposed in rice for LRP development. Using this method, we described the expression dynamic patterns of several genes, especially *OsPINs*, during the rice LRP development and suggested a “fountain” model of auxin transport in LRP of rice, which is similar with that in *Arabidopsis.*

## Methods

### Plant growth condition

Rice seeds (*Oryza sativa* L. cv. Nipponbare) were grown in solution culture (Yoshida et al. [1976]) in controlled-environment room at temperature regimes of 28/22°C (day/night) and 70% humidity under a 12-h photoperiod. One-week-old seedlings were harvest for analysis.

### Dissection of epidermal and ground tissues of primary roots

For dissection of epidermal and ground tissues of rice, the root segments were fixed in FAA (formaldehyde, 95% ethanol and acetic acid in the volume of 10:85:4) solution for at least 24 hours in 4°C. After that, the roots were washed in water for several times and operated carefully under the stereomicroscope (Olympus, Japan) with two needles. With longer time of fixation, the LRP can be separated easier. The procedures of staining, dehydration, clearing, infiltration, and embedding were performed according to Liu et al. ([2005]). The microtome sections were mounted on glass slides for imaging. All the sections showing the patterns of cell divisions in Figure 2H-N have been repeated at least three times.

#### Construction of GUS fusion constructs

For *DR5::GUS* vector construction, the *DR5* element (Ulmasov et al. [1997]) coupled to the CaMV 35S minimal promoter was amplified and digested at the site of *Sal* I and *Bam* H I, and inserted into *pBI101.3*, which carries the structural gene for GUS and the terminator sequence of the nopaline synthase (*NOS*) gene. The primers used in PCR amplification were GTCGACGGTATCGCAGCCCCCTTTTGTC and GGATCCTCCCTGTAATGTAAATAGTAAT. For *OsSHR1::GUS* vector construction, the promoter of *OsSHR1* was amplified by PCR and digested at the site of *Hin* d III and *Kpn* I, then inserted into *pBI101.3*. The primers used in PCR were TCAAAGCTTCATAACATATGGATCAATACAAAC and TCAGGTACCTAAGCAACGGCGACGAGGAGGA. Construct containing GUS reporter controlled by promoters of *OsPIN1* c, *OsPIN1b* and *OsPIN2* were constructed before (Wang et al. [2009]). These constructs were transformed into the Wild-type (Nipponbare) via *Agrobacterium tumefaciens* EHA105.

#### Histochemical analysis and GUS assay

Histochemical GUS analysis was performed according to Jefferson et al. ([1987]). Transgenic plant root samples were incubated in X-gluc solution at 37°C. After staining, the tissues were rinsed and fixed in FAA.

## Authors’ original submitted files for images

Below are the links to the authors’ original submitted files for images. Authors’ original file for figure 1 Authors’ original file for figure 2 Authors’ original file for figure 3

## Abbreviations

LRP: Lateral root primordia:
PIN: PIN-FORWMED:
OL: Out layer:
IL: Inner layer:
GUS: β-glucuronidase:
SHR: SHORT-ROOT:
