# Supplementary figures and images for: Histological characterization of the lateral root primordium development in rice
Source: Bot Stud. 2014 May 10;55:42. doi: 10.1186/s40529-014-0042-x (PMC5432738; doi:10.1186/s40529-014-0042-x)

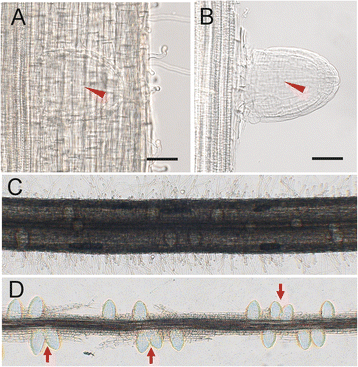

Supplement: Supplementary file 1 — Authors’ original file for figure 1 [file 40529_2014_42_MOESM1_ESM.gif]

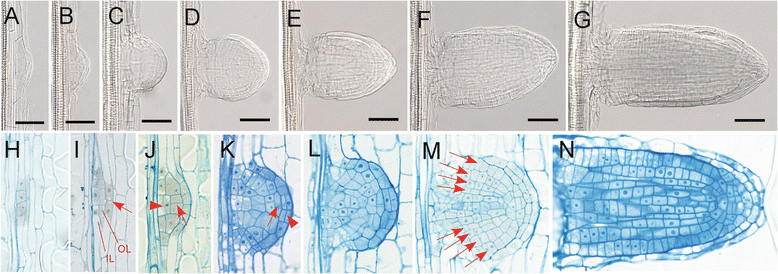

Supplement: Supplementary file 2 — Authors’ original file for figure 2 [file 40529_2014_42_MOESM2_ESM.gif]

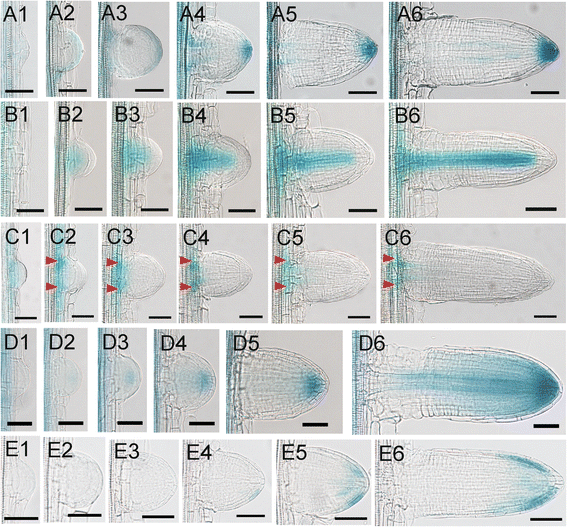

Supplement: Supplementary file 3 — Authors’ original file for figure 3 [file 40529_2014_42_MOESM3_ESM.gif]
